# Improving Mechanical Properties of PVPPA Welded Joints of 7075 Aluminum Alloy by PWHT

**DOI:** 10.3390/ma11030379

**Published:** 2018-03-05

**Authors:** Guowei Li, Furong Chen, Yongquan Han, Yahong Liang

**Affiliations:** School of Materials Science and Engineering, Inner Mongolia University of Technology, Hohhot 010051, China; liguowei-2008@imut.edu.cn (G.L.); hyq@imut.edu.cn (Y.H.); liangyahong@imut.edu.cn (Y.L.)

**Keywords:** PVPPA welding, heat treatment, 7075 aluminum alloy, microstructure, mechanical properties

## Abstract

In this study, 7075 aluminum alloy with a thickness of 10 mm was successfully welded with no obvious defects by pulsed variable polarity plasma arc (PVPPA) welding. The mechanical properties of PVPPA welded joints have been researched by post weld heat treatment (PWHT). The results indicate that the heat treatment strongly affects the mechanical properties of the welded joints. The tensile strength and the microhardness of the welded joints gradually improved with the increase of the solution temperature. With the increase of the solution time, the tensile strength, and microhardness first dramatically increased and then decreased slightly. The best tensile strength of 537.5 MPa and the microhardness of 143.7 HV were obtained after 490 °C × 80 min + 120 °C × 24 h, and the strength was nearly 91.2% of that of the parent metal, and increased about 35% compared with as-welded. The improvement of strength and microhardness was mainly due to the precipitation of η′ phase.

## 1. Introduction

Al-Zn-Mg-Cu aluminum alloys have been widely used in the spacecraft and industrial fields due to their usefulness in providing lightweight structure, high strength-to-weight ratio, high strength, high toughness, corrosion resistance, etc. [1,2]. Most of the structural components in machines, pressure vessels, transport vehicles, earthmoving equipment, etc., are made by welding method. The welding of 7-series aluminum alloys is difficult for researchers because of the porosity, slag inclusion, and cracks that are easily exhibited in the weld metal zone (WMZ). Also, the loss in mechanical properties as compared to the base material is very significant. These factors make the joining of these alloys by conventional welding processes unattractive [3,4].

Compared with other welding processes, high weld quality and high productivity can be obtained by variable polarity plasma arc (VPPA) welding. The advantages of VPPA welding are attributed mainly to the fully penetrated keyhole-mode welding, where the hydrogen cannot be trapped in the pool, tenacious oxide film can be removed from the workpiece surface, and better fluidity of the metal can be guaranteed in the weld pool [5]. VPPA welding has been used successfully in fabricating a space shuttle’s external tank which is made from aluminum alloy [6,7]. 

Based on VPPA welding, a new method of PVPPA welding has been developed in our previous work, where pulsed current was added in VPPA welding and showed an obvious improvement of strength [8]. The cycling of welding current from a high level to a low level was conducted at a regular PVPPA welding frequency. The other advantage of PVPPA welding is that the current can be controlled by a square wave with a long positive time and short reversed time, and the welding heat can be adjusted by changing the positive current and reversed current. Thus, PVPPA welding was adopted in this study.

For welding of metals, as is well known, the tensile strength and elongation of as-welded joints are lower than those of the base metal. Fortunately, many researchers have found that the mechanical properties of welded joints can be improved by PWHT in some metals [9,10]. Wang et al. (2017) [11] researched the effects of aging treatments on the mechanical properties of Al-Cu-Li alloy joints and found that the strength coefficient of the joints increased from 0.64 to 0.90 after post weld double aging treatment. Fadaeifard et al. (2016) [12] found that the hardness in all zones increased after PWHT and the elastic modulus improved from 69.93 to 81 GPa in gas tungsten arc welded AA6061-T6 alloy. Lin et al. (2017) [13] investigated the microstructure evolution after PWHT and the cryogenic fracture toughness of AA2219 variable polarity tungsten inert gas (VPTIG) welding joints, and the results showed that the strength improved while the plasticity and fracture toughness decreased slightly after PWHT. Therefore, PWHT should be an efficient way to improve the mechanical properties of welded aluminum alloy. 

However, to the knowledge of the authors, there has been no study on the effect of PWHT on the PVPPA welded joints of 7075 aluminum alloy. Therefore, the objective of this study is to investigate the effect of PWHT on a PVPPA welded joint of 10 mm thick 7075 aluminum alloy plate. 

## 2. Experimental

The experimental material was 7075 aluminum alloy (7075-T651). The welding wire was ϕ1.2 mm ER5183 aluminum magnesium alloy. The chemical compositions of the 7075 aluminum alloy (supplied by the Southwest Aluminum (Group) Co., Ltd., Chongqing, China) and 5183 welding wire (supplied by Zheng Zhou ChuanWang Welding Consumables, Zhengzhou, China) are listed in Table 1. Table 2 shows the mechanical properties of the 7075 aluminum alloy. 

**Figure 1:**
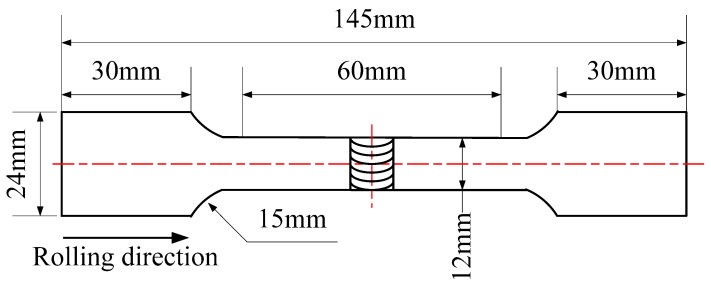
Schematic of tensile specimen.

The welding samples were cut to dimensions of 100 × 80 × 10 mm, and the edge of the specimen was welded L-S (longitudinal-short). Before welding, the surfaces of the samples were sanded using 1000 grit SiC paper, followed by washing away oil pollution in acetone liquid. The VPPA-300 welding power supply was used in the experiment. Pure (99.9%) argon gas was used as the shielding gas and plasma gas. The welding parameters are listed in Table 3. The PWHT parameters are listed in Table 4. The schematic of the tensile specimen is shown in Figure 1. The Chinese GB/T 2651-2008 was adopted in the experiment. Detailed weld microstructural examination was carried out for the cross-sections of the generated welds using a SN-3400 scanning electron microscope (SEM, Hitachi Limited, Tokyo, Japan), phase analysis of the welded joint was completed on a D/Max 2500 X-ray diffractometer (XRD, Rigaku Corporation, Tokyo, Japan), and the composition was analyzed by a 51-XMX1023 energy spectrometer (XES, Hitachi Limited, Tokyo, Japan). The distribution of the second phases and the micro-area composition in the welded joint were observed on a Tecnai G2 F20 transmission electron microscope (TEM, FEI Company, Hillsboro, OR, USA). The tensile tests were carried on a microcomputer-controlled electro-hydraulic servo universal testing machine (ShangHai SANS Measuring instrument manufacturing Co., Ltd., Shanghai, China). The Vickers microhardness was measured by a HXD-1000™ microhardness tester (Shanghai Taiming Optical Instrument Co. Ltd., Shanghai, China) with load 200 g and time 15 s.

## 3. Results

### 3.1. Microstructure

#### 3.1.1. As-Welded

**Figure 2:**
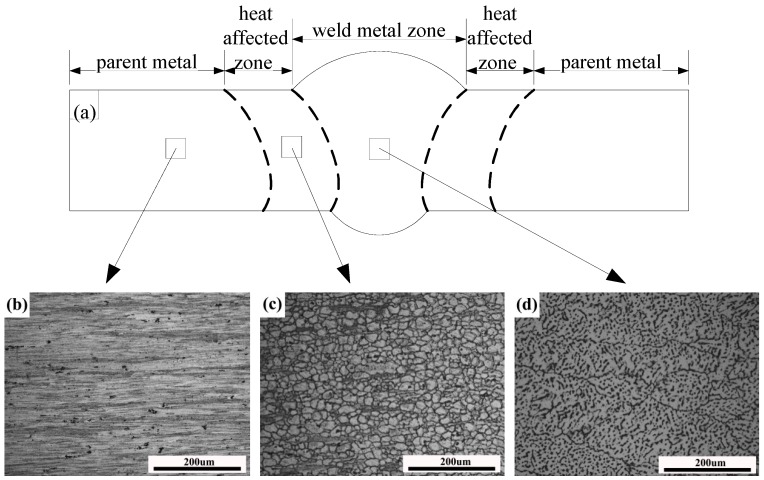
Optical microstructures of welded joint: (**a**) Schematic illustration of the welded joint; (**b**) parent metal; (**c**) heat affected zone; and (**d**) weld metal zone.

**Figure 3:**
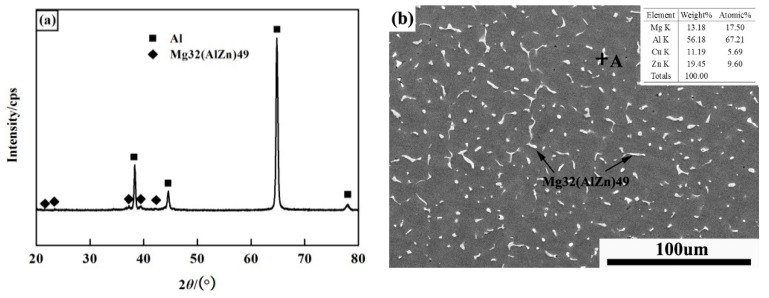
XRD pattern (**a**) and BSE micrograph (**b**) of the welded joint.

The schematic illustration and metallographic microstructures of the corresponding zone on the welded plate are shown in Figure 2. Figure 2a is the schematic illustration of the welded joint. Figure 2b is the microstructure of the parent metal, where it can be seen that the rolling microstructure of the parent metal is unchanged after welding. Figure 2c shows the equiaxed grain shape of the heat affected zone (HAZ), which indicates that the full recrystallization is affected by the welding heat. The microstructure of the WMZ mainly consists of columnar dendrites, as shown in Figure 2d. The phase composition of the welded joint was identified by XRD, and the result is shown in Figure 3a. It can be seen that the phase composition is mainly composed of *α*-Al and *T* (Mg_32_(AlZn)_49_) phase of the as-welded condition. The morphology of the *T* phase, as analyzed by BSEM (Backscatter Scanning Electron Microscope), was distributed in the interdendritic microstructure and exhibits a mainly dotted and strip-shaped appearance, as the white positions shown in Figure 3b. The white positions (Section A) was analyzed by EDS (Energy Disperse Spectroscopy), and the results were shown in Figure 3b. The results of the XRD analysis and the phase composition of Al, Zn, Mg, and Cu elements, confirmed that the bright phase is *T* (Mg_32_(AlZn)_49_) phase. 

#### 3.1.2. Effect of Solution Temperature

**Figure 4:**
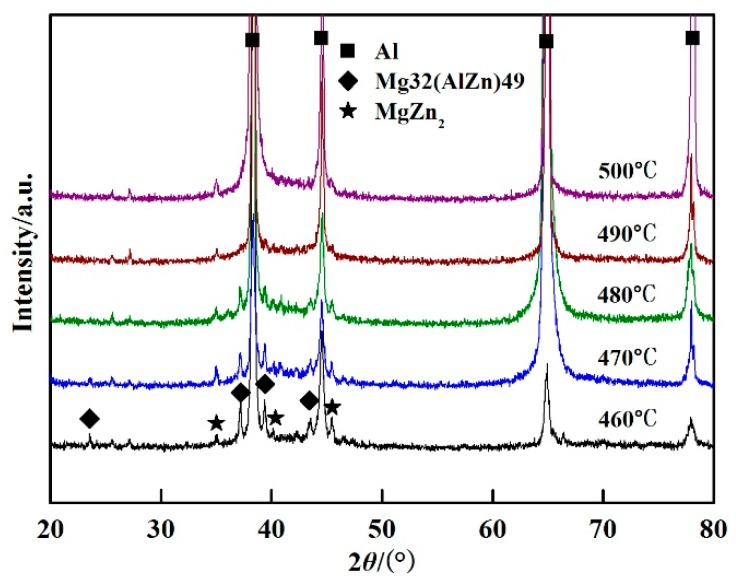
XRD patterns of the welded joints after PWHT with solution at different temperatures for 60 min followed by aging at 120 °C for 24 h.

**Figure 5:**
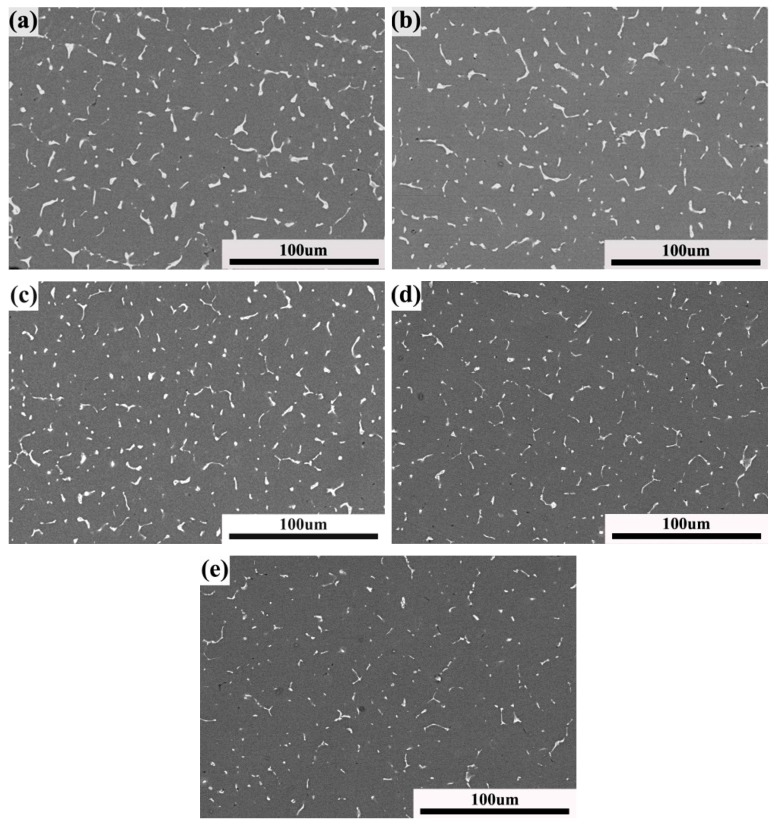
BSE micrographs of welded joints after PWHT with solution at different temperatures for 60 min followed by aging at 120 °C for 24 h: (**a**) 460 °C; (**b**) 470 °C; (**c**) 480 °C; (**d**) 490 °C; and (**e**) 500 °C.

The effect of the solution temperature on the microstructure was researched. Figure 4 shows the X-ray diffraction patterns of the welded joints after PWHT, with the solution at different temperatures for 60 min followed by aging at 120 °C for 24 h. The peak value of the *T* phase decreased with the increase of the solution temperature. In other words, the content of the *T* phase reduced with the increase of the solution temperature. The direct evidence is seen in Figure 5, which shows the BSE micrographs of the welded joints after PWHT. It can be seen that the volume fraction and the size of *T* phase decreased with the increase of the solution temperature. Figure 4 and Figure 5 indicate that the more *T* phase dissolved in the matrix with the increase of solution temperature. With the solution temperature over 490 °C, the volume fraction and the size of *T* phase have hardly changed. The η′ (MgZn_2_) phase precipitated in matrix during the PWHT, as can be seen in Figure 4.

#### 3.1.3. Effect of Solution Time

**Figure 6:**
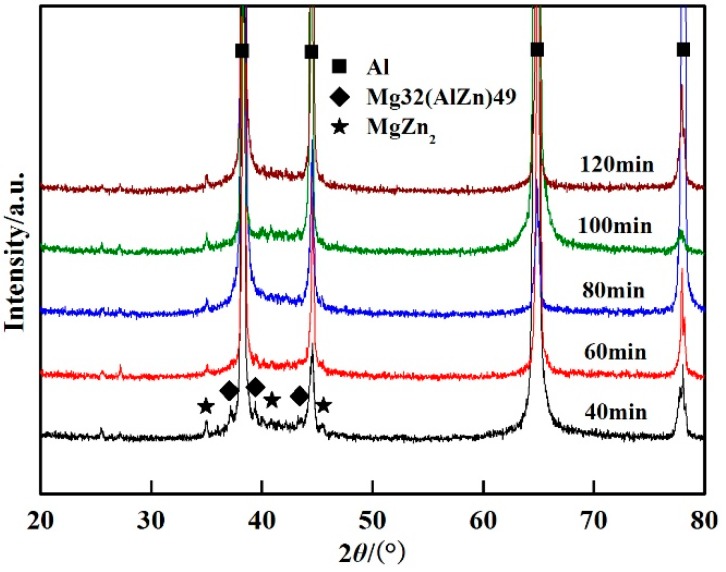
XRD patterns of the welded joints after PWHT with solution at 490 °C for different times followed by aging at 120 °C for 24 h.

**Figure 7:**
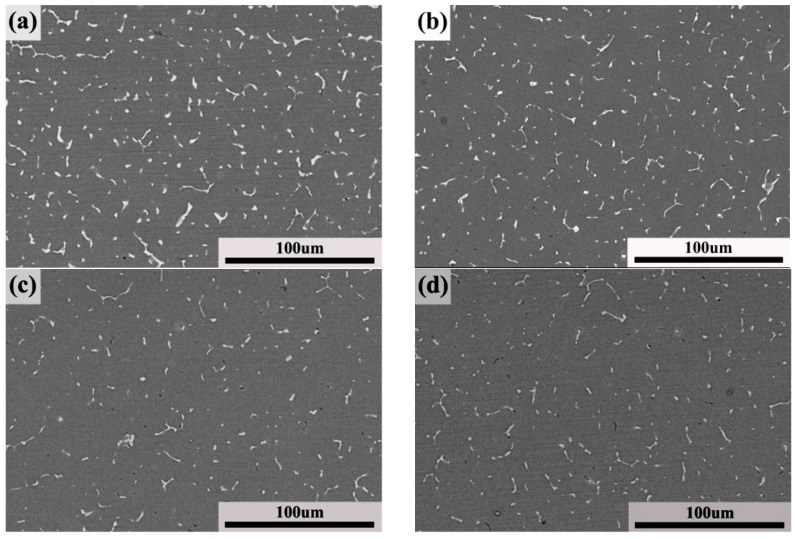

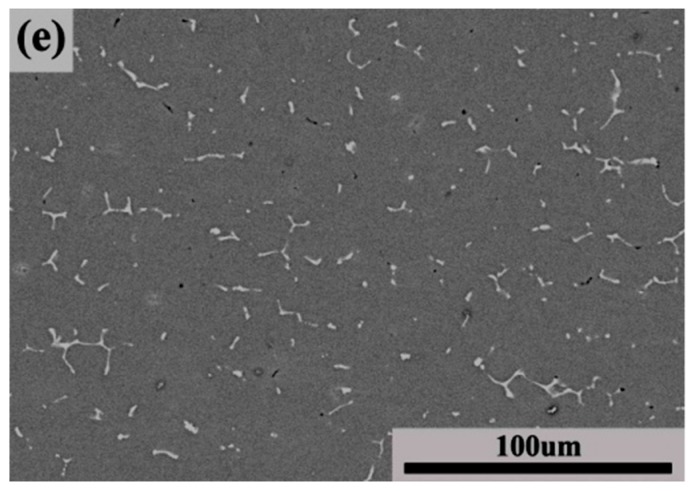
BSE micrographs of welded joints after PWHT with solution at 490 °C for different times followed by aging at 120 °C for 24 h: (**a**) 40 min; (**b**) 60 min; (**c**) 80 min; (**d**) 100 min; and (**e**) 120 min.

The effect of the solution time on the microstructure was also studied. Figure 6 shows the X-ray diffraction patterns of the welded joints after PWHT with the solution at 490 °C for different times followed by aging at 120 °C for 24 h. The phases are mainly composed of *T* phase and η′ phase. The corresponding BSE micrographs of the welded joints after PWHT are shown in Figure 7. It can be seen from Figure 6 and Figure 7 that the content and the scale of the *T* phase decreased with the increase of the solution time. However, the volume fraction and scale of the *T* phase hardly changed when the solution time was over 80 min, as can be seen from Figure 7.

### 3.2. Mechanical Properties

#### 3.2.1. Effect of Solution Temperature

**Figure 8:**
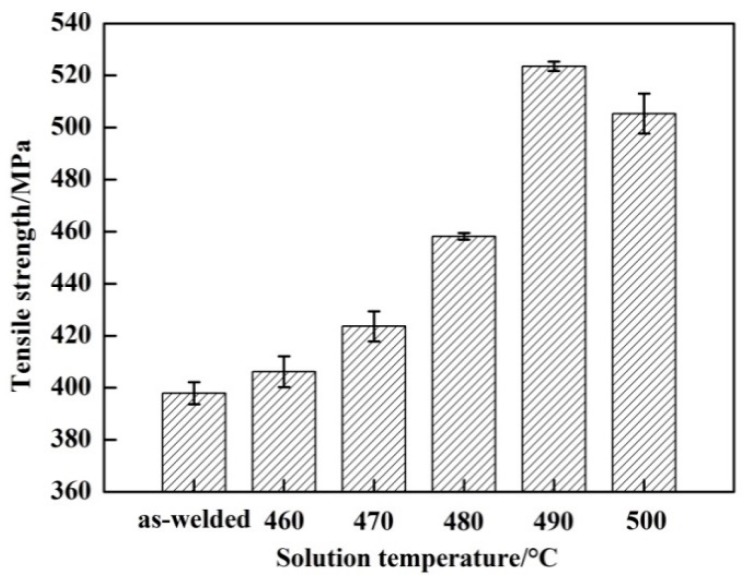
Tensile strengths of welded joints between as-welded and PWHT with solution at different temperatures for 60 min followed by aging at 120 °C for 24 h.

Figure 8 shows the effect of the solution temperature on the tensile strength of the welded joints. The tensile strength of the as-welded joint is 397.9 MPa, which is about 67.5% of the parent metal. During PWHT, the tensile strength increased to the peak value and then decreased with the increase of the solution temperature. The tensile strength of the welded joint is 406.2 MPa with the solution temperature of 460 °C, which is slightly higher than as-welded. With the continued increase of the solution temperature, the tensile strength of the welded joint gradually improved. The highest tensile strength of 523.5 MPa was obtained at 490 °C, which is nearly 88.8% of the parent metal, and it was increased by 31.6% compared to the untreated welded joint. However, the tensile strength of the welded joint with a solution temperature of 500 °C decreased slightly compared with 490 °C. Figure 8 indicates that the tensile strength of the welded joint is evidently improved by PWHT. The same trend was also observed in the results for the microhardness of the welded joints.

**Figure 9:**
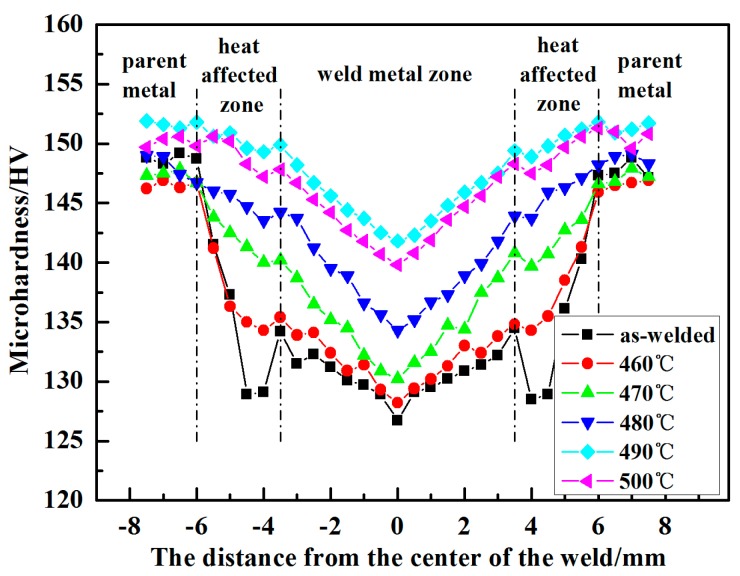
Microhardness distribution of welded joints between as-welded and PWHT with solution at different temperatures for 60 min followed by aging at 120 °C for 24 h.

Figure 9 shows the microhardness distribution of the welded joints. The microhardness distribution of the as-welded joint in Figure 9 presents a typical W shape. The center of the joint exhibits the minimum value of microhardness, and the HAZ of the welded joint has an obvious trough of microhardness. The PWHT has a significant effect on the joint. First, the microhardness changes from a W shape to a V shape, where the microhardness gradually decreased from the parent metal to the center of the welded joints after PWHT. After PWHT, the microhardness of the welded joint was enhanced obviously. As a matter of fact, 7075 aluminum alloy is a precipitation-strengthened alloy. The microhardness could be enhanced by the MgZn_2_ phase which precipitated in matrix during the aging process, and led to the increased microhardness of the HAZ. Secondly, the microhardness of the welded joint was improved greatly by PWHT. Taking the microhardness of the center, for example, the microhardness of as-welded is about 126.7 HV, while the highest value of 141.8 HV is achieved by the solution treated at 490 °C for 60 min followed by aging at 120 °C for 24 h. Finally, the microhardness of the welded joints first increased and then decreased during the PWHT, and the highest microhardness was obtained when the solution temperature was 490 °C.

#### 3.2.2. Effect of Solution Time

**Figure 10:**
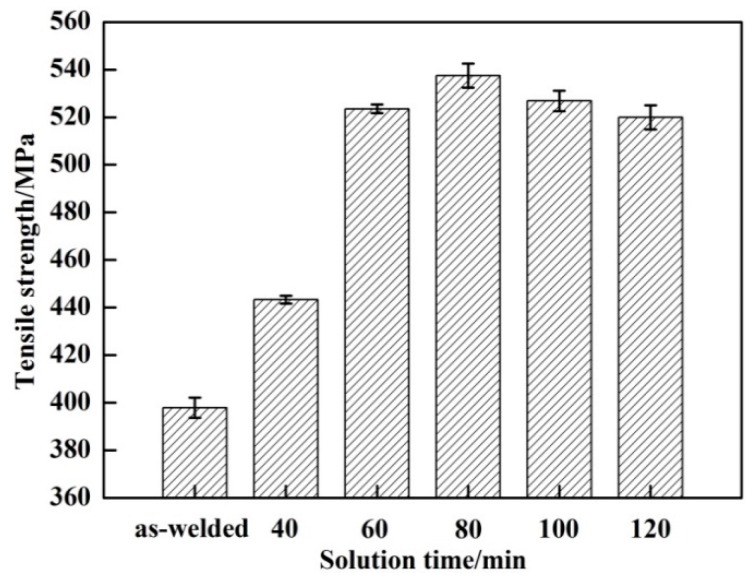
Tensile strengths of welded joints between as-welded and PWHT with solution at 490 °C for different time followed by aging at 120 °C for 24 h.

Figure 10 shows the effect of the solution time on the tensile strength of the welded joints. The tensile strength of the welded joints was improved evidently by PWHT. With the increase of the solution time, the tensile strength first dramatically increased and then decreased slightly. The tensile strength of the welded joint is 443.4 MPa with the solution time of 40 min, which is slightly higher than as-welded. With the increase of the solution time, the tensile strength of the welded joint gradually improved. The highest tensile strength was about 537.5 MPa at 80 min, which is nearly 91.2% of the parent metal, and about 139.7 MPa higher than as-welded. When the solution time was over 80 min, the tensile strength decreased slightly, but was still significantly higher than as-welded. 

**Figure 11:**
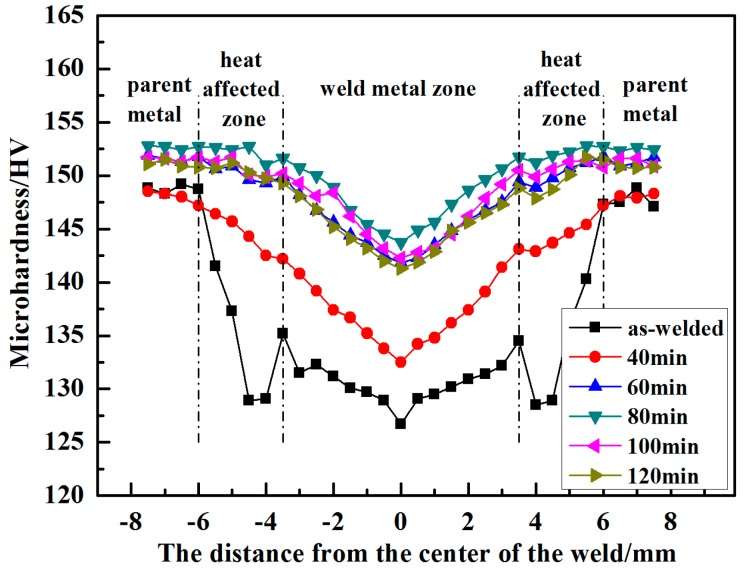
Microhardness distribution of welded joints between as-welded and PWHT with solution at 490 °C for different times followed by aging at 120 °C for 24 h.

Figure 11 shows the results of the microhardness of the welded joints after PWHT with the solution at 490 °C for different times followed by aging at 120 °C for 24 h. As can be seen from Figure 11, the microhardness of the welded joint was improved evidently by PWHT, exhibiting a V shape on the figure. The microhardness first increased and then decreased with the increase of the solution time. The maximum value of 143.7 HV at the center of the welded joints was obtained at 80 min, which is about 17 HV higher than as-welded. With the solution time over 80 min, the microhardness decreased slightly.

## 4. Discussion

The weldability of high strength aluminum alloy is very poor, and defects such as hot cracking, porosity, and other welding defects often appear in welded joints, limiting their extensive application. The preferred welding processes of 7075 aluminum alloy frequently use a gas tungsten arc welding or laser welding process due to their comparatively easy application [14], but it needs to be welded two or more times when the aluminum is thicker, and the cost of the laser welding equipment is high. This study finds that 7075 aluminum alloy with a thickness of 10 mm can be successfully welded with no obvious defects using PVPPA welding. Usually, the tensile strength of the welded joint reaches 60% of the parent metal when using the traditional welding method. In this work, the tensile strength of the welded joint by PVPPA welding reached 67.5% of the parent metal. Therefore, high weld quality and high productivity can be obtained by PVPPA welding in one stroke.

**Figure 12:**
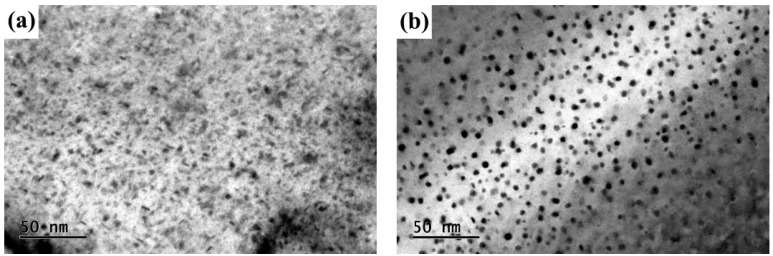
Comparison of the TEM micrographs of welded joints between as-welded and PWHT with solution at 490 °C for 80 min followed by aging at 120 °C for 24 h: (**a**) as-welded and (**b**) 490 °C, 80 min.

However, as with other methods, the tensile strength of the welded joint by PVPPA welding is poor. In order to improve the strength of the welded joint even more, the researchers looked for other methods, and PWHT was found to be an effective method for improving the mechanical properties of welded joints [15,16,17], and now confirmed by our results. As shown in Figure 8 and Figure 9, the tensile strength and the microhardness were enhanced by PWHT. The reason for the improvement of the mechanical properties of the welded joint can be concluded as the result of precipitation strengthening. Figure 12a shows the TEM image of the as-welded joint. It can be seen that a small amount of unclear precipitated phase precipitated in the as-welded matrix, possibly because the welded joint has insufficient time to precipitate due to the faster cooling rate after welding. Figure 12b shows the TEM image of the welded joints of PWHT at 490 °C for 80 min followed by aging at 120 °C for 24 h. It can be seen that the nanoscale precipitated phase was distributed uniformly in the matrix, and the quantity increased obviously compared to the as-welded.

**Figure 13:**
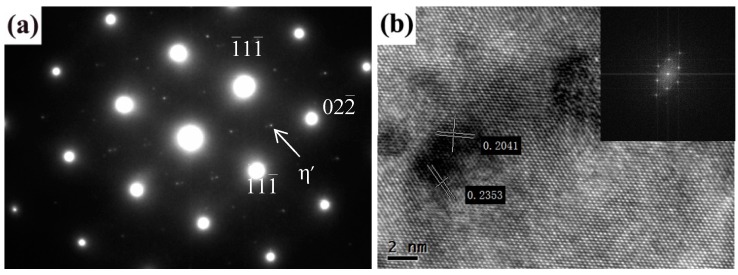
TEM images of welded joints after PWHT with solution at 490 °C for 80 min followed by aging at 120 °C for 24 h: (**a**) SAED image and (**b**) HRTEM image.

Figure 13 shows the TEM images corresponding to Figure 12. The presence of η′ is confirmed by examination of the SAED patterns on [011]_Al_ (as shown in Figure 13a). The high resolution images of the weld after Fourier transform are shown in Figure 13b, and the interplanar distance d is calculated as corresponding to the d value of MgZn_2_. Therefore, the precipitated phase in Figure 12b can be determined as the MgZn_2_ phase. Dispersion precipitation is the main reason for the enhancement following heat treatment [18,19,20].

The solution temperature and time exhibited an important effect on the mechanical properties of the welded joints. As shown in Figure 8, the tensile strength and microhardness dramatically increased as the solution temperature reached 490 °C, and the maximum values of the tensile strength and microhardness were obtained. During the PWHT, the *T* phase dissolved in the matrix, and the content and scale of the *T* phase decreased with the increase of the solution temperature. This means that the content of Mg and Zn solute elements increased in the matrix and resulted in more precipitation of MgZn_2_ during the aging heat treatment. Therefore, the greater content of MgZn_2_ led to the higher mechanical properties. With the solution temperature over 490 °C, the *T* phase showed no change, as shown in Figure 5. The solution time also had the same effect on the mechanical properties of the welded joints. The tensile strength and microhardness dramatically increased as the solution time reached 60 min, and the maximum value of the tensile strength and microhardness was obtained at 80 min. With the continued increase of the solution time, the tensile strength and microhardness changed little. The changing of the *T* phase during PWHT can explain why the volume fraction and the scale of the *T* phase decreased with the increase of the solution time. With the solution time over 80 min, the *T* phase was unchanged, as shown in Figure 7. With the continued increase of the solution temperature and time, the tensile strength and microhardness showed little change. However, the slight decrease of the tensile strength and microhardness may be due to grain growth [21].

## 5. Conclusions

The 7075 aluminum alloy was successfully welded by the PVPPA welding with no obvious defects. The microstructure and mechanical properties of PVPPA welded joints have been investigated by PWHT. The major conclusions can be summarized as follows:

(1) 7075 aluminum alloy with a thickness of 10 mm can be successfully welded with no obvious defects by PVPPA welding. The tensile strength of the welded joint using PVPPA welding reached 67.5% of the parent metal.
(2) The solution temperature and time have an important effect on the mechanical properties of the welded joint. During PWHT, the tensile strength and microhardness first increased and then decreased slightly with the increase of the solution temperature and solution time. The change of the mechanical properties was due to the dissolution of the *T* phase and the precipitation of η′ phase during PWHT.
(3) The mechanical properties of the welded joint using PVPPA welding can be improved by the PWHT. The highest tensile strength of 537.5 MPa was obtained when the solution was treated at 490 °C for 80 min followed by aging at 120 °C for 24 h, which is about 91.2% of the parent metal. The maximum microhardness (143.7 HV) at the center of welded joints was also obtained under this heat treatment.

## Figures and Tables

**Table 1:** Chemical compositions of 7075 aluminum alloy and ER5183 welding wire (wt-%).

	Elements	Zn	Mg	Cu	Cr	Mn	Ti	Fe	Si	Impurity	Al
Materials											
7075		5.1∼6.1	2.1∼2.9	1.2∼2.0	0.18∼0.28	0.3	0.2	0.5	0.4	0.15	Bal.
5183		0.25	4.3∼5.2	0.1	0.05∼0.25	0.5∼1.0	0.15	0.4	0.4	0.15	Bal.

**Table 2:** Mechanical properties of 7075 aluminum alloy.

Alloy	Tensile Strength (MPa)	Yield Strength (MPa)	Elongation (%)	Hardness (HV)
7075	589.2	535.3	12	150

**Table 3:** Welding parameters of PVPPA.

**Welding Parameters**	**Positive Polarity Current (A)**	**Reversed Polarity Current (A)**	**Welding Speed (mm·min^−1^)**	**Wire Feeding Speed (mm·min^−1^)**	**Plasma Gas Flow Rate (L·min^−1^)**
value	240∼260	280∼300	150	220	2.0
**Welding Parameters**	**Protection Gas Flow Rate (L·min^−1^)**	**Tungsten Electrode Neck-In (mm)**	**Time Ratio (ms)**	**High Frequency (Hz)**	**Low Frequency (Hz)**
value	15	3	21:4	50	1

**Table 4:** Parameters of PWHT.

Number	Solution Temperature (°C)	Solution Time (min)
1	460	60
2	470	
3	480	
4	490	
5	500	
6	490	40
7		60
8		80
9		100
10		120
